# Spatiotemporal dynamic evolution and driving factors of desertification in the Mu Us Sandy Land in 30 years

**DOI:** 10.1038/s41598-020-78665-9

**Published:** 2020-12-10

**Authors:** Xueying Han, Guangpu Jia, Guang Yang, Ning Wang, Feng Liu, Haoyu Chen, Xinyu Guo, Wenbin Yang, Jing Liu

**Affiliations:** 1 College of Desert Control Science and Engineering, Inner Mongolia Agricultural University, Hohhot, 010018 China; 2 Institute of Desertification Studies, Chinese Academy of Forestry, Beijing, 100091 China

**Keywords:** Environmental impact, Natural hazards

## Abstract

The Mu Us Sandy Land is located in the middle of the farming pastoral ecotone of northern China. The direction of the development of desertification has a direct impact on the economy and development of the northern region. Six remote sensing images acquired during 1990–2017 served as data sources. Using an ENVI 5.3 and ArcGIS 10.3 platform an analysis was conducted of the dynamic changes nearly 30 years in desertified land using a center of gravity moving model, annual change rate, a transfer matrix, and an aeolian desertification index; the factors driving desertification were discussed. The research shows that the time period can be divided into three stages of desertification: development (1990–2000), rapid reversal (2000–2010), and stable reversal (2010–2017). A total of 1680 km^2^ of desertification were managed over the three stages. Spatially, the distribution of the center of desertification from west to east includes mild, moderate, severe, and extreme desertification, which is consistent with the spatial distribution trends of desertified land in the Mu Us Sandy Land. By the end of 2017, the degree of desertification of the Mu Us Sandy Land was in the central area > northwest > southwest > east > south. Nearly 30 years, the wind speed has decreased year by year at the rate of 0.1 m s^−1^, which directly reduce the ability to winds to transport soil in the Mu Us Sandy Land and promoted the reversal of desertification. From 1990 to 2010, the climate tended to become warmer and drier. Environmental protection policies along with human intervention and control of desertification have played important roles in reversing desertification. From 2010 to 2020, under the general background of a warm-wet climatic tendency, rational use of sand resources and strengthening scientific control of desertification inducing factors are the keys to reversing desertification.

## Introduction

Desertification, also known as wind erosion desertification^1^, is one of the main forms of desertification^2^, which occur mainly in arid, semi-arid, and partially semi-humid areas. Desertification is a process of land degradation caused by excessive human activities and the improper use of resources^3–5^. Desertification, as an important environmental and socio-economic problem, creates perplexing problems for the world’s social and governmental agencies^6^. Desertification affects one quarter of the world’s land area, two thirds of the world’s countries, and nearly one billion people^7,8^ with China being one of the most seriously affected counties^9^. According to the fifth survey of desertification in China, desertification caused by wind erosion affects 1.8 million km^2^, accounting for 69.9% of China’s desertified land area; these areas are mainly located in northwest, north, and northeast sandstorm areas in China^10,11^. Typical areas of desertification in China are concentrated in these three northern regions. Sandstorms affecting farmland result in low and unstable crop yield. The direct economic losses caused by the serious degradation of desertified pastures amounted to 4.5 billion yuan a^−1^. Without the control of desertification in these “three northern” areas, the ecology and the living standard of the people in China will not be fundamentally improved. Therefore, controlling the distribution and driving factors of desertified land in typical areas of northern China is very important.

When conducting desertification research, one of the most important aspects is the dynamic monitoring and evaluation of desertified land^5^. Traditional desertification monitoring mostly relies on national macro-control, taking provinces and cities as units, carrying out field measurements and other means, which has the disadvantages of being time- and labor-consuming, requiring long-term monitoring, being inefficient, and so on^12,13^. With the emergence and development of remote sensing technology, its advantages of economy, objectivity, and macro-scale monitoring make up to a certain extent the deficiencies of traditional desertification monitoring and research methods^14–18^, and it has the advantages of providing rapid data updating, detailed information content, a wide range of observation, high accuracy, and so on^8^. In the early days, China made use of remote sensing technology and computer simulation to compile a “Desertification Indicator Handbook”^19^, the United Nations Food and Agriculture Organization’s “Provisional Methods for Desertification Assessment and Mapping”^20^, the “Field Survey Handbook on Land Degradation”^21^, and the “World Atlas of Desertification”^22^. In addition, Wang et al.^23^, Wu et al.^24^, Duan et al.^25^, and other scholars have used remote sensing imagery to carry out dynamic monitoring research on land desertification in China, enriching the research results related to land desertification using 3S technology in China.

As early as the late 1950s, Zhu often talked about the problem of desertification, believing that the 530,000 hm^2^ of what was considered sandy in the Mu Us Sandy Land in Shaanxi and Inner Mongolia’s Yikezhao League was mostly converted into desert due to human intervention, i.e. anthropogenic desert^5^. The book “Natural Conditions and Their Improvement and Utilization in the Mu Us” published in 1983 evaluates the natural environment in this region and its impact on the occurrence and development of desertification, and puts forward a comprehensive plan to prevent and control desertification, which is of great practical significance in guiding practice^26^. In 1992, experts from the United Nations Educational, Scientific, and Cultural Organization analyzed the global environment and identified the Mu Us Sandy Land as one of the world’s ninth most environmentally sensitive areas^27,28^. This region is located in a typical ecological transition zone^29^, with significant ecological vulnerability and spatial heterogeneity^30^, and has been attracting extensive attention from scholars worldwide. Wang and Li^31^ used 1986–1987 Landsat Thematic Imager (TM) imagery and topographic maps to investigate the distribution of quicksand in the Mu Us Sandy Land^31^; Wu et al*.*^24^ made use of TM images acquired in spring of 1987 and 1993 to carry out dynamic monitoring on desertified land in the Mu Us Sandy Land. The results show that the desertified area in 7 years research areas had decreased by 1,936 km^2^^24^. Hao et al*.*^32^, in his research on desertification and land use in the Mu Us region, concluded that land desertification in this region was still in a state of development from the mid-1980s to the end of 1990s, and pointed out that unreasonable land use patterns and intensity of human land use were the important factors leading to land desertification in the Mu Us region^32^. Using Landsat TM/Operational Land Imager (OLI) imagery acquired from 1990 to 2014, Liu et al*.*^33^ studied the changes of landscape pattern in the Mu Us Sandy Land. The results showed that the fixed and semi-fixed sandy land areas in the Mu Us Sandy Land increased, the mobile sandy land area decreased significantly, the human activities expanded rapidly, and the climatic factors had little influence on desertification^33^. Guo et al*.*^34^ analyzed the spatial distribution and dynamic change process of desertified land in different stages in the Mu Us Sandy Land and in surrounding areas. The results showed that the spatial extent of desertified land in the Mu Us Sandy Land and the surrounding areas showed a continuous downward trend, and the conditions in desertified land were improving^34^. Han et al*.*^28^, Li^35^, Qiao^36^, Bai and Cui^37^, Quan et al*.*^38^, and others have studied desertification in the Mu Us Sandy Land from different angles and have provided useful findings^39,40^.

As an important ecological barrier in north China, the Mu Us Sandy Land can contribute to improving China’s climate by helping to avoid drought in the north and floods in the south, preventing the further spread of desertification, and preventing desertification from moving eastward and southward^41^, which is of great significance to the improvement of China’s environment. Therefore, observing the desertification of the Mu Us Sandy Land over a long time period is very important. Using Landsat TM/OLI imagery, this study of the Mu Us sandy desertified land covering 1990–2017 and the factors driving desertification were analyzed. The dynamic evolution of desertification in time and space in different stages of the Mu Us Sandy Land desertified land area, the annual change trends, change in the center of gravity, and driving forces were analyzed. This was done from three aspects of temporal dynamics, spatial evolution, and driving forces to reveal the spatial and temporal evolution of desertification. The goal was to guide the reasonable use of the research area of desertified land and related areas, which will provide a scientific basis for the optimal allocation of resources and support sustainable development.

## Materials and methods

### Survey of the research area

**Figure 1:**
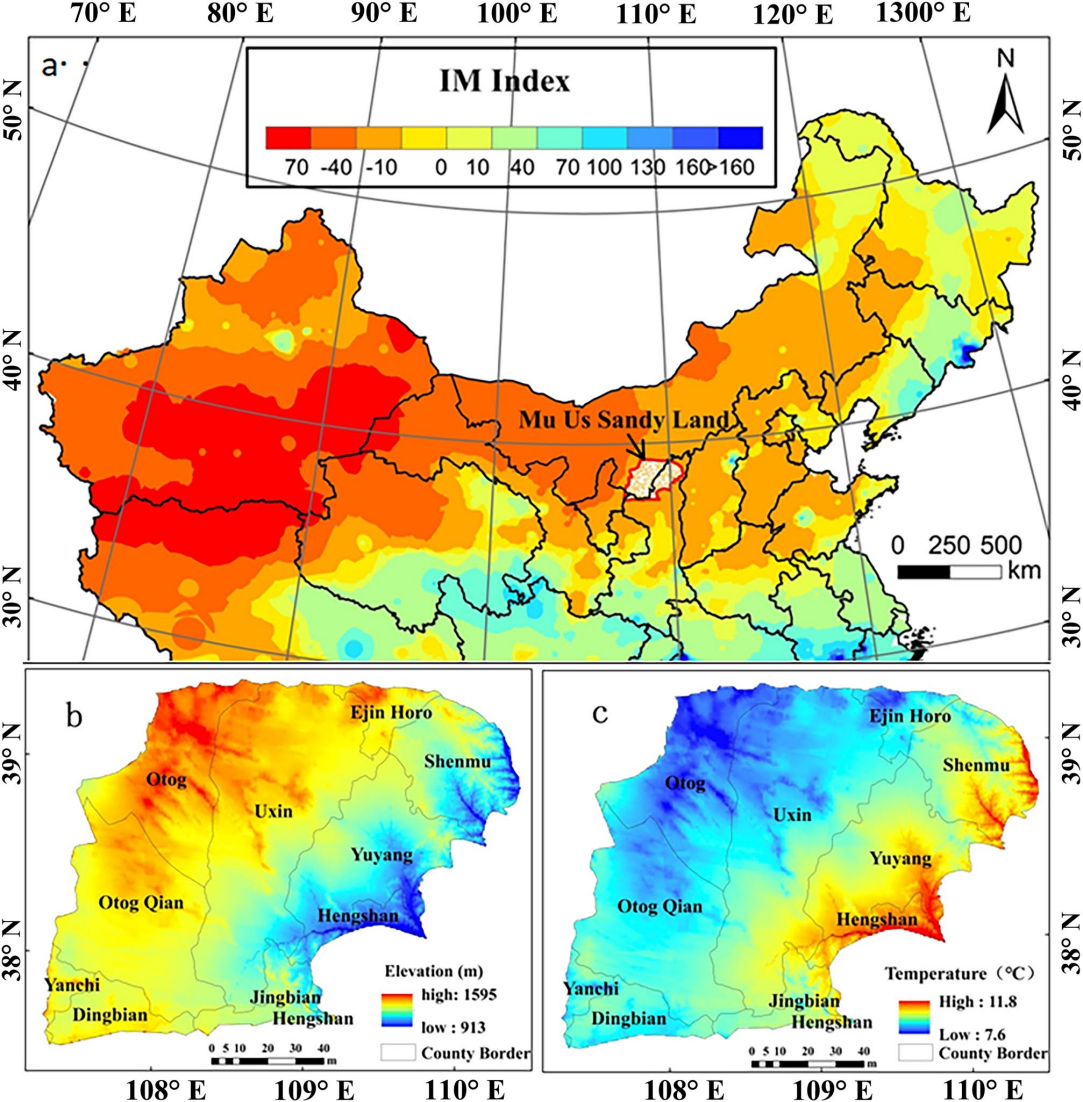
Geographic location map of the Mu Us Sandy Land. (**a**) Wetness index map of Northern China and the Mu Us Sandy Land was based on Xu’s Chinese meteorological background data set^42^. (**a**) Location, (**b**) elevation map was extracted from the elevation of China (http://www.resdc.cn/data.aspx?DATAID=123), and (**c**) distribution of mean annual temperature was derived from the spatial difference data set of China's average temperature in 2015^42^ (http://www.resdc.cn/data.aspx?DATAID=228).

The Mu Us Sandy Land is mainly distributed in the south of the city of Ordos in Inner Mongolia, in the north of the city of Yulin in Shaanxi Province, and in the northeast of Yanchi County in Ningxia Hui Autonomous Region (Fig. 1a)^42^, with a total area of about 42,200 km^2^ at 37.45°–39.37° N and 107.67°–110.5° E. The elevation decreases gradually from west to east (average 1254 m), and the northwest reaches up to 1595 m (Fig. 1b). The Mu Us Sandy Land is located in a transition zone where the climate shifts across an arid–semi-arid–humid region (Fig. 1a). Under the influence of the north Pacific subtropical high, the southeast monsoon prevails in summer. Abundant precipitation forms with a northwestward extension of polar fronts, so the climate is warm and humid. In the half-year-long winter, an anticyclone wind system prevails under the control of Mongolian–Siberian high pressure systems, which is dominated by strong northwest winds, forming a cold and dry climate. Spring and autumn are transitional periods between Mongolian high pressure and Pacific low pressure systems, with the latter typically lasting only a short time. In spring, the temperature rises quickly and the weather becomes dusty, while autumn is cool and short^26^. It has a typical semi-arid continental climate with an average annual temperature of 6.5–10.3 °C, which is higher in the southeast than in the northwest (Fig. 1c). The annual precipitation of 250–440 mm increases from west to southeast. There are 170 rivers and streams in the sandy land, and both surface water and groundwater are abundant. The soil types were mainly chestnut, brown calcium, lime, and sandy soils, but with a low nutrient content. The main vegetation communities include *Artemisia ordosica, Caragana microphylla, Sabina vulgaris,* and others^43^. By 2017, the distribution of the increasing human population in the Mu Us Sandy Land became increasingly prominent and problematic. The population density in the western region is currently far smaller than in the eastern region. In particular, the Yuyang district has the largest population density, 78.0 people/km^2^, which far exceeds the United Nations recommendation for semi-arid zones of 20 people/km^2^^44^; nevertheless, the population density of Uxin Qi, Otog Qi, and Otog Qian Qi banners is relatively small, with the minimum density as low as 4.9 people/km^2^.

### Data sources and methods

#### Data sources

**Figure 2:**
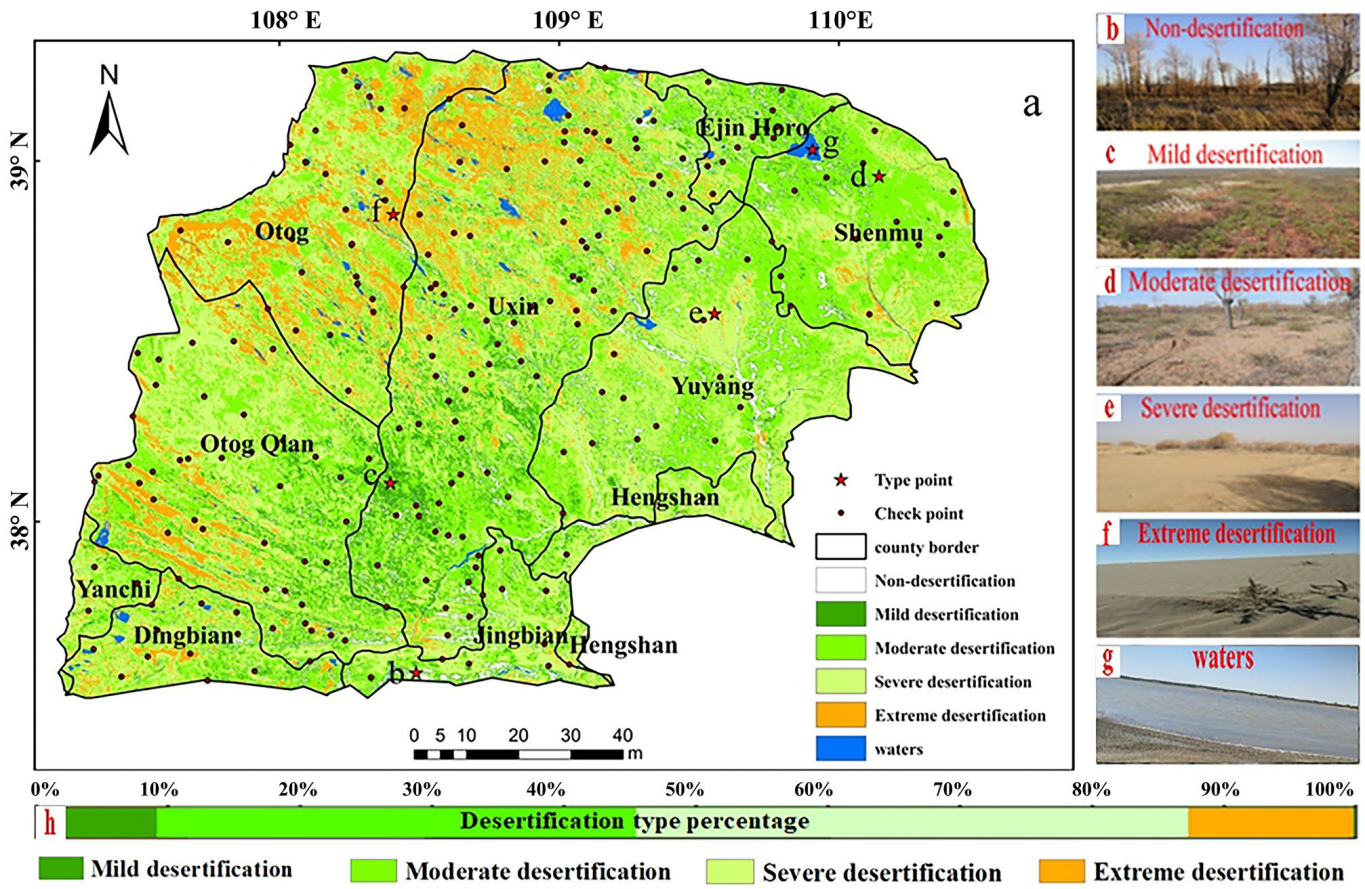
(**a**) Thematic map of the Mu Us Sandy Land in 1995 compiled based on Landsat 5 Thematic Mapper images. Grey circle Check points: based on 2017 desertified land field verification points. Red star Type points: inset photos show typical examples of (**b**) non-desertification, (**c**) mild desertification, (**d**) moderate desertification, (**e**) severe desertification, (**f**) extreme desertification, (**g**) water, (**h**) different desertification type percentage in the study area. The boundary of each flag county in Mu Us Sandy Land is derived from the administrative boundary data of county level in China (http://www.resdc.cn/data.aspx?DATAID=202).

In this study, Landsat TM/OLI remote sensing images (1990, 1995, 2000, 2007, 2010, and 2017) were used as data sources; these data were obtained from the geospatial data cloud (http://www.gscloud.cn/). The six images were acquired in the vegetation growing season of the Mu Us Sandy Land (June to September), which ensured the comparability of images and features. In order to highlight the nature of ground features, bands (4), (3), and (2) were used for standard false color synthesis of Landsat TM images, and bands (5), (4), and (3) were used for standard false color synthesis of Landsat OLI images. The resultant images could clearly distinguish different cover types for subsequent interpretation. Strict geometric and atmospheric corrections were carried out on each image to eliminate the influence of the atmosphere and light on the reflection of ground objects and the non-systematic geometric deformation caused by the instability of the sensor’s own height and attitude. The error after correction was always less than 0.5 pixels. The visual interpretation marks of the Mu Us Sandy Land were obtained through field investigation data and remote sensing interpretation in 2010. The images were interpreted by human–computer interaction using an ArcGIS 10.3 platform; the remote sensing images acquired in 2017 were used to select 350 verification points. The field verification points were targeted at typical and easily confused ground types for verification. If the desertification type was unique to any one point, the density of investigation points could be appropriately reduced; conversely, the verification points could be increased when appropriate (Fig. 2). With an analysis of vegetation coverage, the degree of desertification, quicksand area detailed information such as the proportion of investigation, and through a confusion matrix, it is concluded that the overall classification accuracy was 92.6% with a Kappa coefficient of 91.0%.

The meteorological data (mean precipitation, temperature, and wind speed) used in this study were obtained from the China meteorological science data sharing service (http://cdc.cma.gov.cn/). Data on human factors (population, livestock, and farmland) are available from a series of national statistical yearbooks (http://www.resdc.cn/data.aspx?DATAID=250).

#### Desertified land classification system

According to the monitoring indicators of desertified land in 2000^24^ and the results of the China 973 Project titled “Research on the Process of Desertification and its Prevention and Control”^25^, and referring to a large amount of relevant references^2,7,45^, the desertified land was divided into five types: non-desertified as well as mildly, moderately, severely, and extremely desertified lands (Fig. 2).

#### Annual change rate of desertified land

The annual growth rate of desertified land (*R*) can reflect the development status and interannual change of desertified land in different years and is an important index used to study the dynamic change of desertified land. The *R* was calculated using Eq. (1)^1^:

1 $$\text{R} = \left(\sqrt[{\text{n}}]{\frac{{\text{Q}}_{1}}{{\text{Q}}_{2}}}-{1}\right) \times \text{100,}$$

where *R* is average annual growth rate of desertified land (%), *Q*_1_ and *Q*_2_ are the areas of desertified land in the last year and the initial year of the study period, and *N* is the number of years between start year and end year.

#### Transfer matrix of Desertification land

A transfer matrix was used to analyze the quantitative description of state and state transfer in a research system, which comes from system analysis^46^. A transfer matrix can comprehensively and specifically allow the analysis the quantitative transformation and change structure of various land use types, and its mathematical expression is^47^:

2 $$Sij\hspace{0.17em}=\hspace{0.17em}\left[\begin{array}{c}\begin{array}{cc}{\mathrm{S}}_{11}& {\mathrm{S}}_{12}\\ {\mathrm{S}}_{21}& {\mathrm{S}}_{22}\\ \cdots & \cdots \end{array} \begin{array}{cc}\cdots & {\mathrm{S}}_{1\mathrm{n}}\\ \cdots & {\mathrm{S}}_{2\mathrm{n}}\\ \cdots & \cdots \end{array}\\ \begin{array}{cc}{\mathrm{S}}_{\mathrm{n}1}& {\mathrm{S}}_{\mathrm{n}2}\end{array} \begin{array}{cc}\cdots & {\mathrm{S}}_{\mathrm{nn}}\end{array}\end{array}\right],$$

where *S* is the area, *S*_*ij*_ is the land use status at the beginning and end of the study period, and *n* is the number of land use types.

#### Aeolian desertification index (ADI)

In order to provide a comprehensive analysis of the changes in desertification in the Mu Us Sandy Lands from 1990 to 2017, a quantitative indicator of desertified land—ADI—is used as Eq. (3)^48^:

3 $$\mathrm{ADI}=\frac{\left({S}_{s1}+2{S}_{mo}+3{S}_{se}+4{S}_{\mathrm{ext}}\right)}{\mathrm{SA}},$$

where ADI is the desertification Index and *S*_s1_, *S*_mo_, *S*_se_, *S*_ext_, and SA represent the land areas of mildly, moderately, severely, extremely, and total area of desertified land. Note that ADI values ranged from 0–4, ADI was positively correlated with the degree of desertification, and a larger ADI value reflects more severe desertification in the study area.

#### Desertification land gravity transfer model

Based on the calculation of the barycenter coordinates of different desertified lands, a spatial transfer map of the barycenter of desertified lands in the Mu Us Sandy Land was prepared for this paper. The dynamic spatial and temporal changes of desertified lands were reasonably described by combining the transfer distance of the barycenter of desertified lands with the changes in the direction. The calculation formula of the gravity center coordinates of a certain type of desertified land in year *t* is shown in Eqs. (4) and (5)^25^:

4 $$X_{t} = \sum\nolimits_{i = 1}^{n} {(C_{ti} \times X_{ti} )/} \sum\nolimits_{i = 1}^{n} {C_{ti} } ,$$

5 $$Y_{t} = \sum\nolimits_{i = 1}^{n} {(C_{ti} \times Y_{ti} )/} \sum\nolimits_{i = 1}^{n} {C_{ti} } ,$$

where *X*_t_ is the longitude of a certain type of desertified land in year *t*, *Y*_t_ is the latitude of a certain type of desertified land in year *t*, *N* is the number of patches of the land type of this type of desertification in year *t*, *C*_ti_, is the area of the ith patch in year *t*, *X*_ti_: the geometric center of gravity longitude coordinates of the ith patch of the desertified land type in year *t*, and *Y*_ti_ is the geometric barycentric latitude coordinates of the *i*th patch of the desertified land type in year *t*.

## Results of analysis

### Time change of desertified land

#### Change in desertified land area

The desertified land area of the Mu Us Sandy Land has decreased by 1680 km^2^ in the past 30 years, with an average loss of 60 km^2^ a^−1^ desertified land (Table 1). Mild desertification increased at the speeds of 3.0% × a^−1^. The reversion rates of severe and extreme desertification were 2.7% × a^−1^ and 1.7% × a^−1^, respectively.

**Table 1:** Desertification land area of Mu Us sandy land and its changes in different periods.

Year		Mild desertification	Moderate desertification	Severe desertification	Extreme desertification	Total area (km^2^)/annual change rate (%)
1990	Area/km^2^	4910	15,430	12,810	3040	36,190
1995	Area/km^2^	3400	13,410	15,000	4460	36,270
2000	Area/km^2^	2710	11,980	17,410	4860	36,960
2007	Area/km^2^	6300	19,000	8000	2750	36,050
2010	Area/km^2^	9500	16,410	7270	2020	35,200
2017	Area/km^2^	11,090	15,510	6010	1890	34,500
1990–1995	Area change/km^2^	− 1510	− 2020	2190	1420	+ 80
	Annual change rate/%	− 7.1	− 2.8	3.2	8.0	0.1
1995–2000	Area change/km^2^	− 690	− 1430	2410	400	+ 690
	Annual change rate/%	− 4.4	− 2.2	3.0	1.7	0.4
2000–2007	Area change/km^2^	3590	7019	− 9410	− 2110	− 911
	Annual change rate/%	12.8	6.8	− 10.5	− 7.8	− 0.4
2007–2010	Area change/km^2^	3200	− 2589	− 730	− 730	− 849
	Annual change rate/%	14.7	− 4.8	− 3.1	− 9.7	− 0.8
2010–2017	Area change/km^2^	1590	− 900	− 1260	− 130	− 700
	Annual change rate/%	2.2	− 0.8	− 2.7	− 1.0	− 0.3
1990–2017	Area change/km^2^	6180	80	− 6800	− 1150	− 1690
	Annual change rate/%	3.0	0.0	− 2.7	− 1.7	− 0.2

The year 2000 was the turning point of the dynamic change of the land with different degrees of desertification; mildly desertified land first decreased and then increased; the valley and peak values appeared in 2000 (2710 km^2^) and 2017 (11,090 km^2^), respectively. Moderate desertification showed a trend of decreasing–increasing–decreasing. The valley and peak values appeared in 2000 (11,980 km^2^), and 2007 (19,000 km^2^), respectively. Subsequently, due to the strengthening of the work designed to control desertification, moderate desertification showed a trend of decreasing again. The development trend of both severe and extreme desertification showed that these desertification areas gradually increased from 1990 to 2000, and gradually decreased after 2000.

Based on the annual trends of desertified land (Table 1), the nearly 30 years of desertified land change can be divided into three stages: development, rapid reversal, and steady reversal.

Development period: from 1990 to 2000, the spatial extent of mildly and moderately desertified land decreased year by year, while the spatial extent of severely and extremely desertified land increased. The spatial extent of mildly desertified land decreased at a rate of more than 4.4% × a^−1^, while the spatial extent of areas under severe desertification increased by at least 3.0% × a^−1^, showing a general trend of development. Rapid reversal period: from 2000 to 2010, the spatial extent of severely and extremely desertified land both decreased, resulting in a continuous increase of mildly desertified land at a rate of more than 12.8% × a^−1^. Steady reversal period: from 2010 to 2017, the spatial extent of moderately, severely, and extremely desertified land decreased year by year, while that of mildly desertified land increased; during this time period, the annual rate of change was small, presenting a stable trend of the reversal of desertification.

#### Time transfer of desertified land

**Figure 3:**
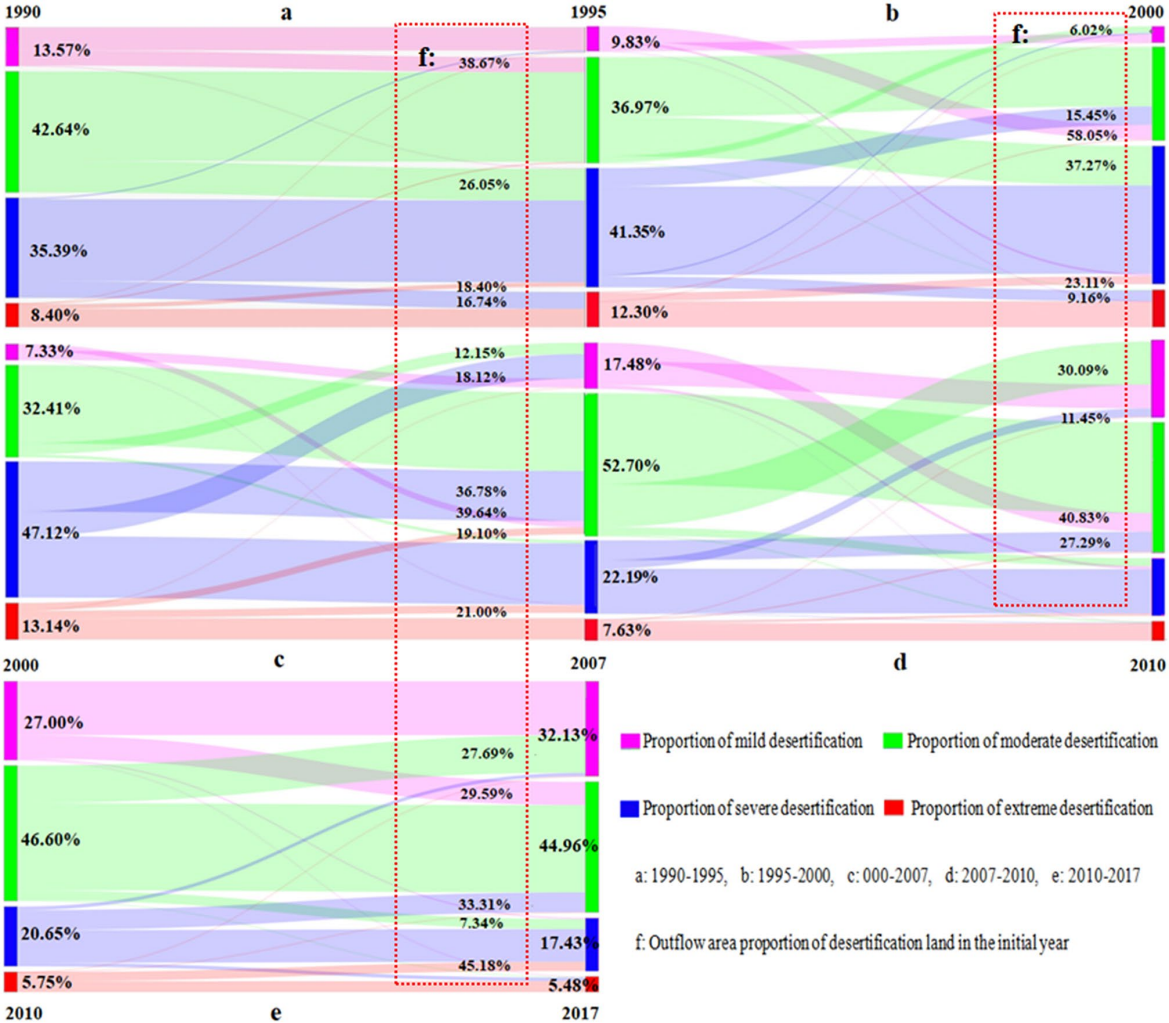
Changes in desertification land area transfer in the study area during: (**a**) 1990–1995, (**b**) 1995–2000, (**c**) 2000–2007, (**d**) 2007–2010, (**e**) 2010–2017, (**f**) the proportion and direction of desertification area outflow in the initial year.

From 1990 to 1995 (Fig. 3a), the level of desertification mainly deteriorated step by step, with new desertification occurring slowly. About 38.7% of the areas of mild desertification developed into moderate desertification (Fig. 3f), 26.1% of moderately desertified land developed into severe desertification, and only 18.4% of extreme desertification reversed the trend to severe desertification. From 1995 to 2010 (Fig. 3b), different degrees of desertification were more active. From 1995 to 2000, 58.1% of mild desertification was transformed into moderate desertification, and 37.3% of moderate desertification deteriorated to severe desertification, which was the main reason for the large increase of the proportions of moderate and severe desertification. From 2000 to 2007 (Fig. 3c), the transformation of some areas of severe and extreme desertification continued. Among them, the proportions of the areas with severe desertification that reverted to mild and moderate desertification were 18.1% and 36.8%, respectively, and those of extreme desertification that reverted to severe and moderate desertification were 21.0% and 19.1%, respectively. From 2007 to 2010 (Fig. 3d), the proportions of areas with moderate and severe desertification that transformed into mild desertification were 30.1% and 11.5%, respectively, which was the main reason for the obvious increase of mild desertification. From 2010 to 2017 (Fig. 3e), the trend of desertification was mainly reversed step by step.

In 1990, the main desertification types were moderate (42.6%) and severe (35.4%). By 2017, the main desertification types had changed to moderate (45.0%) and mild (32.0%), and the degree of overall desertification was significantly reduced. The active degree of desertification in the stages from 1990 to 1995 and from 2010 to 2017 were significantly lower than that in other stages.

#### ADI

**Figure 4:**
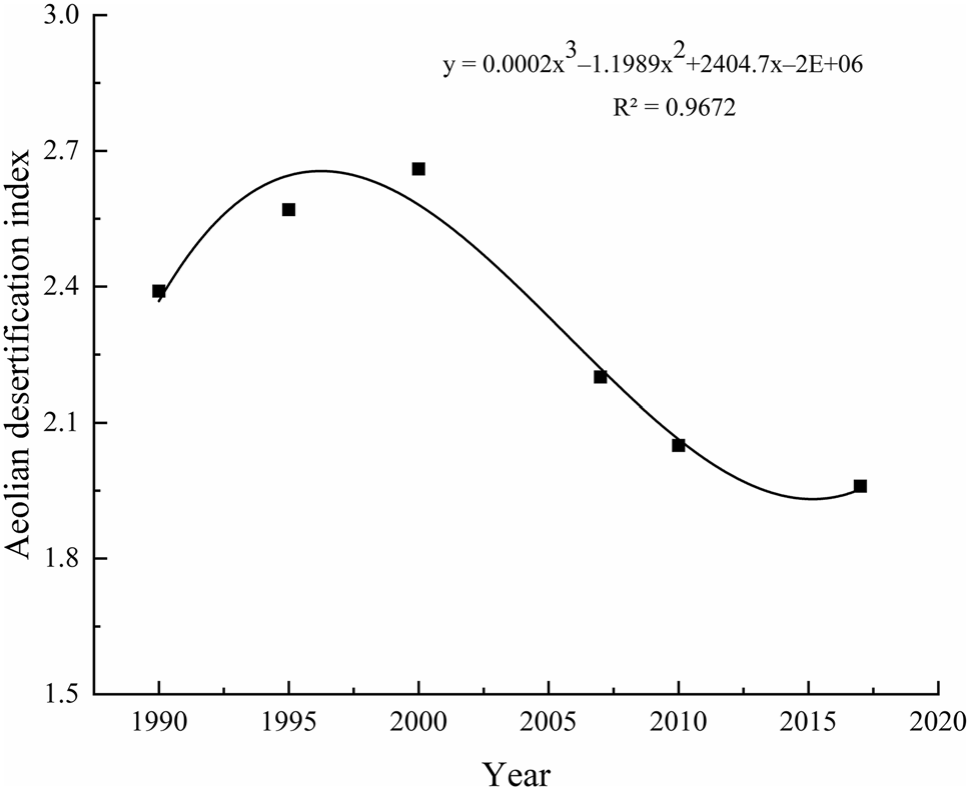
Changes of the desertification index in the study area.

According to the ADI of the Mu Us Sandy Land (Fig. 4), in the past 30 years the dynamic change of desertified land in the Mu Us Sandy Land can be divided into three stages. The period of 1990–2000 is the developmental stage, when ADI increased by 0.3 over 10 years, with an average ADI of 2.5. The period from 2000 to 2010 is a period of rapid reversal of desertification. The ADI decreased by 0.6 in 10 years, with an average ADI of 2.3. The final period of 2010–2017 is a steady reversal phase, with the ADI index varying by less than 0.1 and an average ADI of 2.0 over 7 years. In 2000, the ADI reached peaked at 2.7 when desertification was the most serious, while in 2017, the ADI fell to the lowest point of 2.0. From 1990 to 2017, the degree of desertification was experiencing a trend of reversing while the degree of desertification declined significantly. This is consistent with the dynamic change trends of desertified land in the past 30 years.

Using regression model to analyze the trends of the desertification index, a third-order polynomial fitting equation was obtained: *y* = 0.0002*x*^3^ − 1.1989*x*^2^ + 2404.7*x* − 2E + 06, correlation *R*^2^ = 0.9643, which was extremely significant. Compared with the dynamic change table of desertified land in the past 30 years, the ADI can reflect the dynamic change trend of desertified land more intuitively and accurately.

The temporal dynamics of desertification in the Mu Us Sandy Land are divided: development, rapid reversal, and steady reversal stages, which can be confirmed in the desertification area statistics, annual change pattern, and ADI; therefore, the three stages can reflect the temporal dynamics of desertification in the Mu Us Sandy Land in the past 30 years.

### Spatial evolution of desertified land

#### Desertified land dynamics

**Figure 5:**
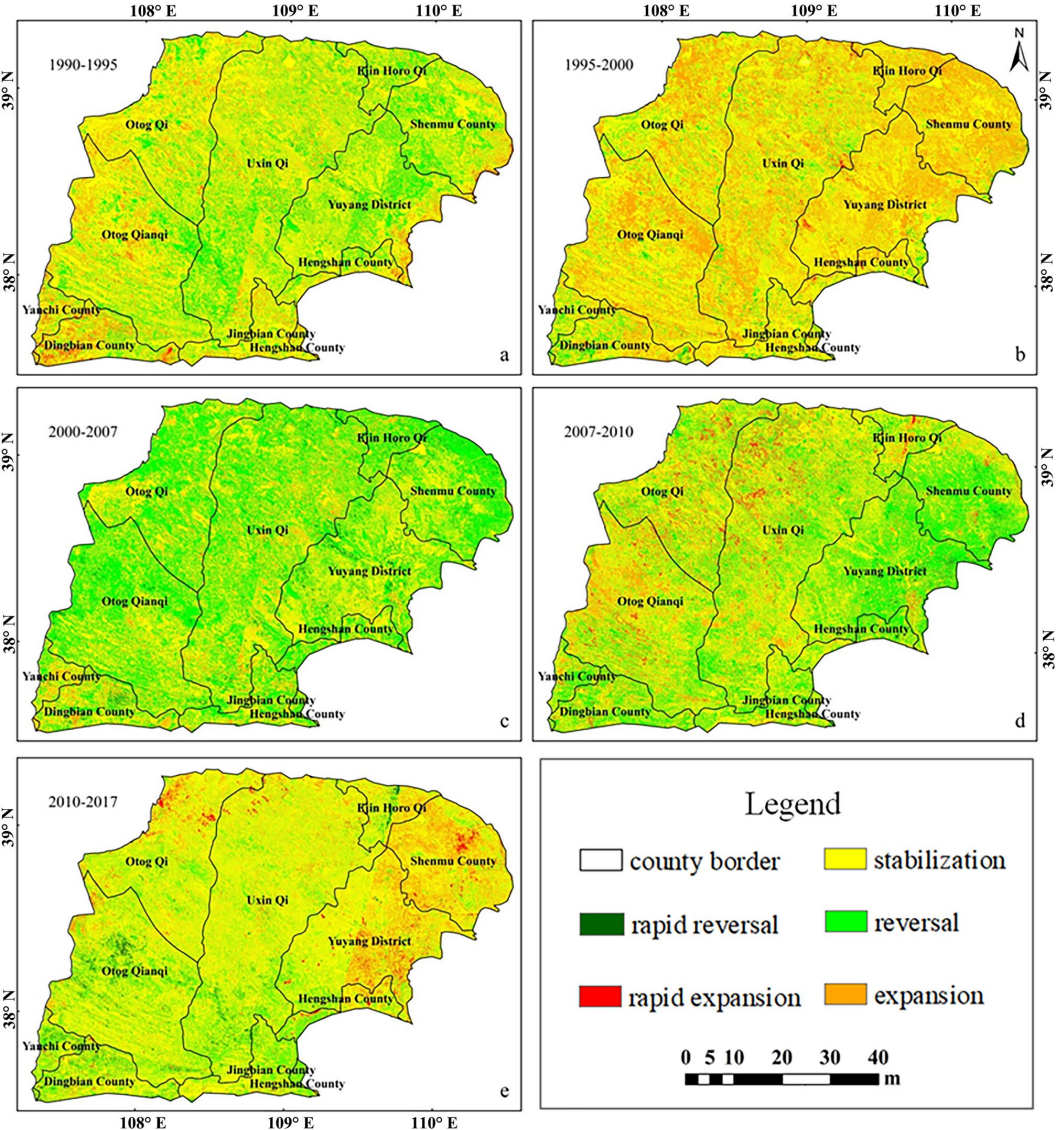
Dynamic changes in desertification in the Mu Us Sandy Land in the last 30 years: changes during (**a**) 1990–1995, (**b**) 1995–2000, (**c**) 2000–2007, (**d**) 2007–2010, (**e**) 2010–2017, legend definitions based on the degree of desertification as follows: rapid reversal (the degree of desertification declined by more than one grade); reversal (declined by one level); stable (degree not changed); expansion of desertification (increased by one level); rapid expansion (increased by more than one level).

From 1990 to 1995 (Fig. 5a), in the eastern and central parts of the Mu Us Sandy Land, 74.4% of desertified land was in a stable state; these areas were mainly in south-central Uxin Qi, Shenmu County, and in most of Yuyang District. In the western part of the Mu Us Sandy Land, 22.5% of the desertified land was in a state of expansion, mainly in the northern part of Otog Qi and the northern part of Otog Qian Qi. Meanwhile, 0.1% of the desertified land in southern Uxin Qi was expanding rapidly. When 1995 to 2000 were compared with the previous period, the spatial extent of land under a stable state of desertification was reduced by 11.3%, while the spatial extent of land where the desertification conditions were reversal increased by 9.5% (Fig. 5b). But the reversal desertification of the land area was still 50.0% of the development area, the overall state of development. From 2000 to 2007 (Fig. 5c), the improvement in areas of desertification was most obvious (a total of 44.2%). These areas were mainly distributed in most of the northwestern part of the Mu Us Sandy Land. The land area in the state of expansion was only 6.6%, distributed in Uxin Qi and the southern edge of the Mu Us Sandy Land. From 2007 to 2010 (Fig. 5d), 683 km^2^ (1.8%) of desertified land existed in the northwestern part of the Mu Us Sandy Land was serious expansion, and 32.3% of desertified land was in an improving condition, among which Yanchi and Shenmu counties were experiencing a rapid reversal of desertification. From 2010 to 2017 (Fig. 5e), the central and southern parts of the Mu Us Sandy Land desertification was mainly stable and improving. Deterioration occurred in the northern part of the Otog Qi, Shenmu County, and in the eastern part of Yuyang district, but the overall situation was improving.

From the perspective of spatial evolution, desertification was in a state of development and expansion from 1990 to 2000, and this situation reversed after 2000. The degree of desertification in the Mu Us Sandy Land showed an overall trend of improvement after 2000, but serious local deterioration and desertification was still occurring. Extreme desertification occurred mainly in the central and western regions. The most severe desertification occurred in the western and marginal areas of The Mu Us Sandy land, and was accompanied with extreme desertification in the course of development. The reversal of mild and moderate desertification showed that the south was superior to the north and the east was superior to the west.

Extremely severe expansion of desertification occurred from 1990 to 2000, mainly on the sandy land in the Midwest (Otog Qi, south of Otog Qian Qi, north of Uxin Qi). Severe expansion of desertification was mainly concentrated on the edges of the western and eastern regions, with only a small increase in mild desertification observed in southern Jingbian County, Ejin Horo Qi, and northern Shenmu County. The degree of desertification is relatively serious, showing the rate of the reversal of desertification was less than the rate of development. The rate of desertification was creating relatively serious problems, showing an overall development trend where the spatial extent of improving land area is less than the areas where desertification was become worse. After 2000, this situation improved, showing that areas with provide conditions of desertification outweighed the loss of land areas to desertification, but the situation was still in a state of fluctuation. From 2000 to 2007, areas of moderate desertification increased significantly in the central and western sandy areas, while mild desertification increased mainly in the northern parts of Otok Qianqi, as well as the northern and eastern marginal areas of sandy areas. The situation related to desertification had obviously reversed, but from 2007 to 2010, extremely severe and severe desertification both rebounded slightly in the central and western regions. From 2010 to 2017, extremely severe and severe desertification also rebounded in Yuyang District and Shenmu County in the east.

#### Migration of the desertification center of gravity

**Figure 6:**
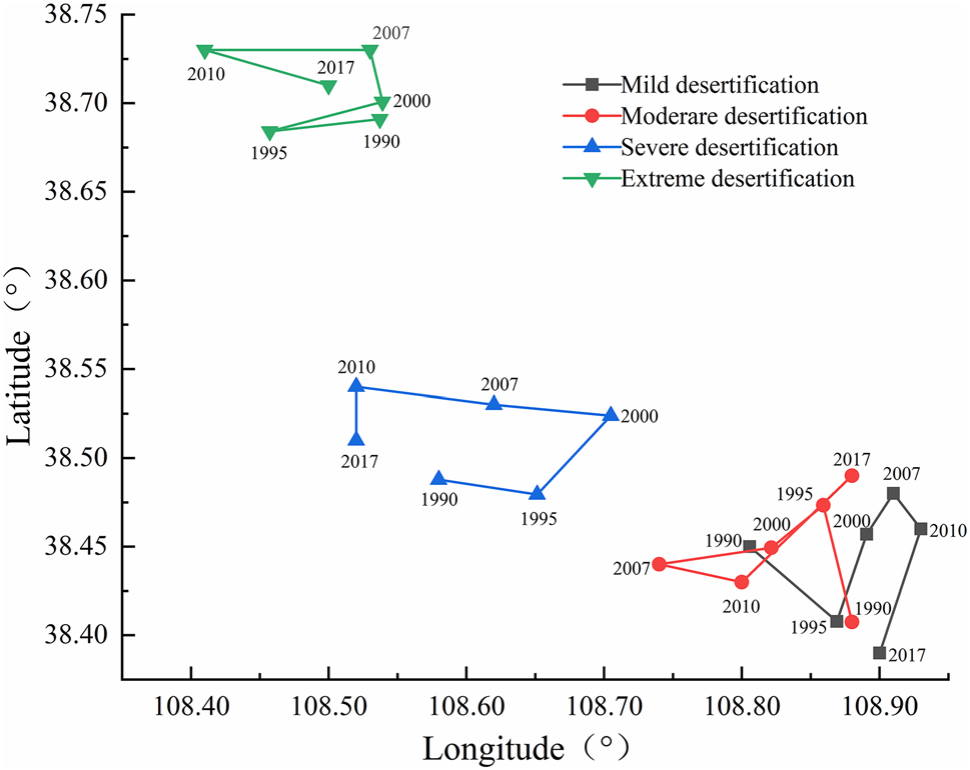
Migration of the centers of gravity of mild, moderate, severe, and extreme degrees of desertification in the study area.

In the past 30 years, the centers of gravity of desertified land in the Mu Us Sandy Land from east to west documented changes in mild, moderate, severe, and extreme desertification, which is consistent with the distribution characteristics of natural conditions (Fig. 6). From 1990 to 2017, the center of gravity for extreme desertification land remained relatively stable and moved only 3 km to the northwest, while the center of gravity for severely desertified land moved 8.80 km to the northwest. The center of gravity for moderately desertified land shifted to the north by 5 km while the center of gravity for mildly desertified land extended 10 km to the southeast. The distribution of land with moderate desertification was similar to that of land with mild desertification, which are both prone to transformation. Therefore, the direction of land change development as it relates to lands with mild and moderate desertification can be controlled by human interference, which should be given great importance in the process of the control and planning of land management.

### Analysis on the driving force of desertification in the Mu Su Sandy land

#### The influence of climatic factors on desertification

**Figure 7:**
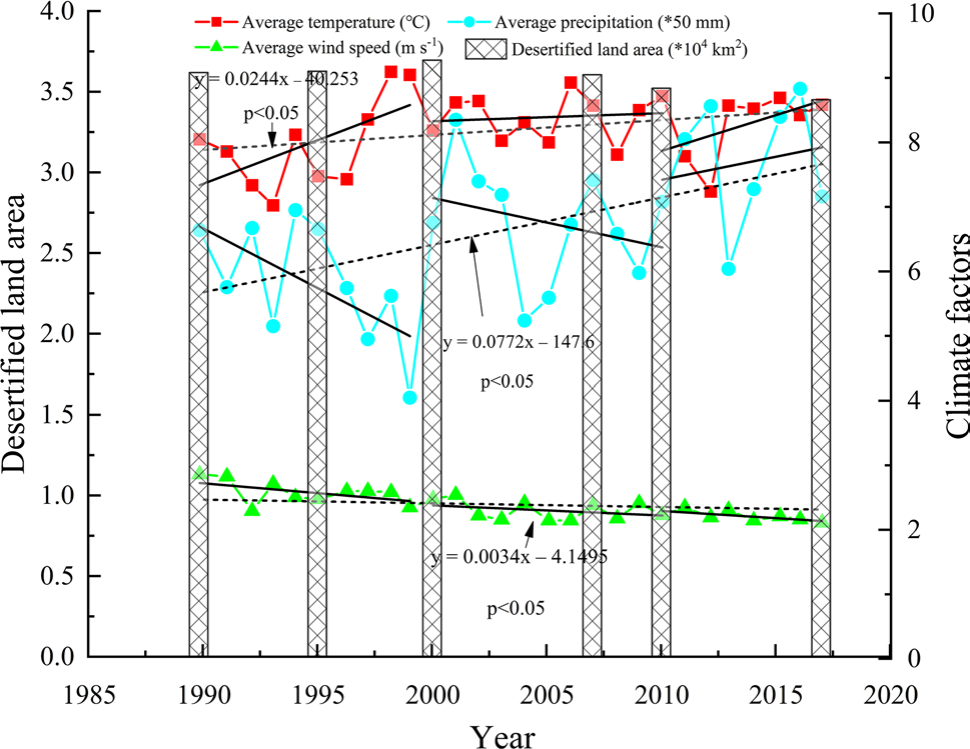
Time series of annual average wind speed, annual average temperature, annual precipitation and desertified land area in the Mu Us Sandy Land from 1990 to 2017.

This paper analyzes the effects of average temperature, precipitation, and wind speed of 1990–2017 on desertification; the influence of these natural factors on desertification of the landscape can be seen in Fig. 7. During 1990–2010, temperatures tended to increase while precipitation was on the decline. The climate tended to become warm-dry, which promoted land desertification. However, the spatial extent of desertified land tended to decreased after an initial increase; other factors led to the development of desertified land in a way that was contrary to changes in climate conditions, the influence of climatic factors on the expansion of desertified land had gradually weakened. From 2010 to 2017, temperature and precipitation both tended to increase year by year, and the climate developed a tendency toward becoming warm-wet. The increases in temperature and precipitation played a positive role in the growth of vegetation and an increase in vegetation coverage, thus promoting the reversal of desertification. The trends in the average annual wind speed decreased by 0.1 m s^−1^ year by year. Regions with higher elevations in the study area, especially in the western region, have a relatively loose soil structure that is easily affected by strong winds. As wind speeds decreased, the erosion carrying capacity decreased year by year as a direct result, promoting the restoration of desertified areas. In the last 30 years, the trend of a moderating climate was basically the same as that of moderating land desertification, where climate is an important factor affecting the development of land desertification. The reduction of wind speed and the favorable combination of temperature and precipitation provide a favorable guarantee for slowing the process of desertification in the Mu Us Sandy Land.

#### Human factors

**Figure 8:**
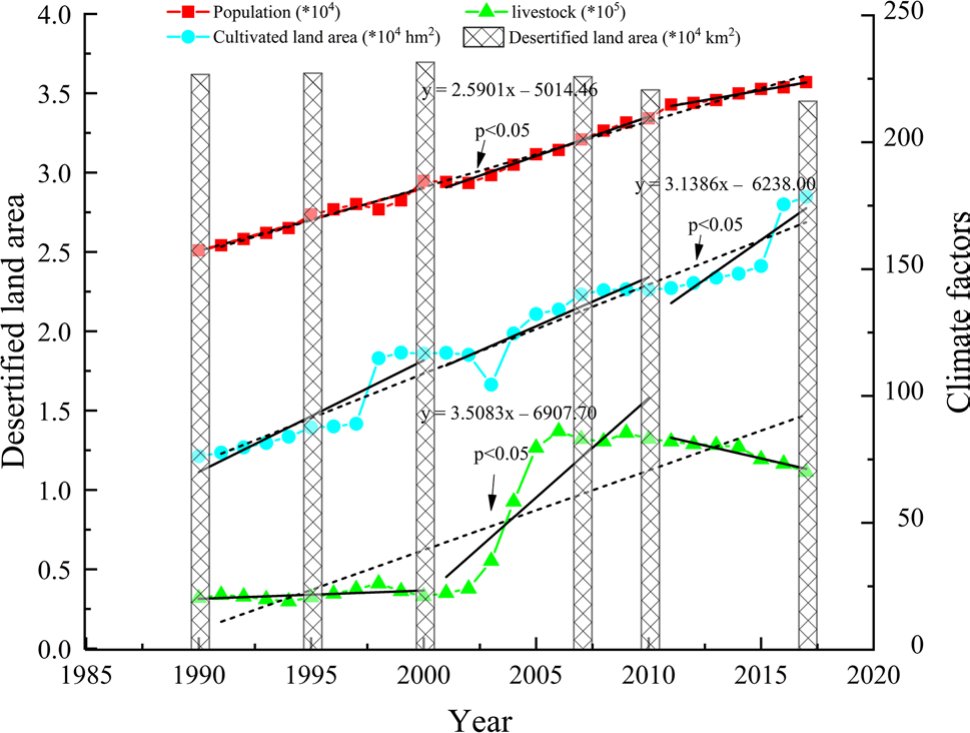
Time series of annual population, livestock production, cultivated land area, and desertified land area of the Mu Us Sandy Land from 1990 to 2017.

In the present study, human population, livestock production, and cultivated land area were analyzed to study the effects of human factors on desertification (Fig. 8). From 1990 to 2017, the population increased by an average of 2.4 × 10^4^ persons a^−1^ for the entire study area; from 1990 to 2000, the population growth rate was 2.5 × 10^4^ persons a^−1^. By 2000, the population density in the Mu Us Sandy Land was 43.8 persons km^2^, which was 2.1 times of the optimal maximum population density in a desertified area as recommended by the United Nations. From 1990 to 2000, the quantity of livestock fluctuated little, with an average increase of 2.2 × 10^5^ head from 2000 to 2010, showing a downward trend after 2010. The large human population of the Mu Us Sandy Land led to pressure to produce food and livestock, resulting in an excessive use of land resources. Excessive use led to more desertification which in turn led to a decrease in land productivity. This became a vicious circle with increasing population pressure. The spatial extent in cultivated land area showed an increasing trend in the past 30 years and increased the most in 1997. In 2001, the Inner Mongolia Autonomous Region implemented the policy of returning farmland to forest (or grassland). The spatial extent of cultivated land showed a decreasing trend from 2001 to 2003, and a growth trend which slowed down after 2010. Obviously, the adjustment of land use structure and other human factors such as the implementation of policy measures are very important to the wise management of desertification problems.

## Discussion

**Figure 9:**
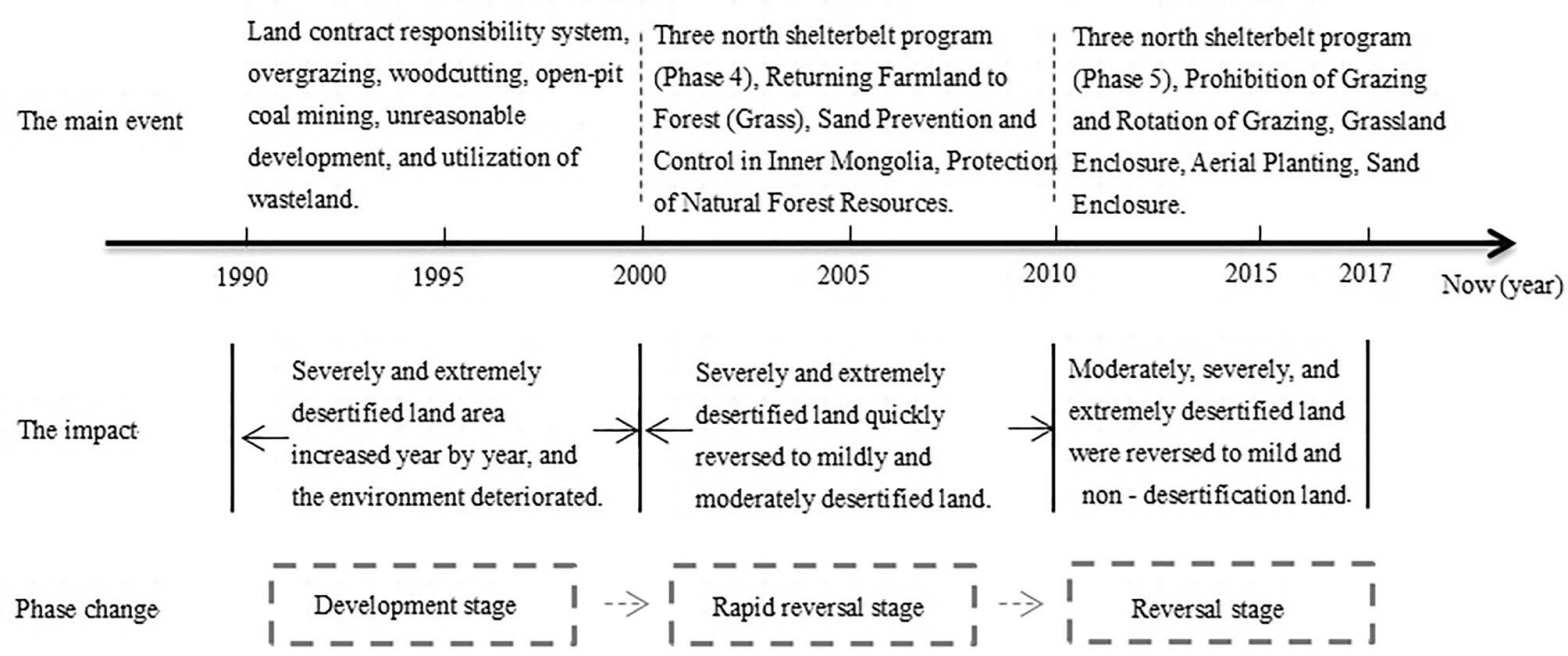
Timeline of human activities in the Mu Us Sandy Land from 1990 to 2017.

Before 2000, the desertified land area of the Mu Us Sandy Land was in the development stage and expanding spatially; a rapid reversal stage of desertification occurred from 2000 to 2010, followed by a slower stable reversal stage of desertification after 2010 (see Fig. 9). The year 2000 was the turning point for land desertification in the Mu Us Sandy Land as described above with desertification initially developed and then reversed which was consistent with the results obtained by Yan and Wu^14^ and Jia et al*.*^49^. In the past 30 years, the desertification of land has been reversed on a total of 1680 km^2^, which is in a general state of reversal of desertification, although the landscape still has some regions with seriously deterioration and desertification (Fig. 5d,e). The control of desertification is still a slow and arduous task. The degree of desertification gradually decreased from west to east in the study area, which was consistent with changes in natural conditions, but ran counter to the effects of human activities^50^. The migrations of the center of gravity for areas with mild and moderate desertification were similar; these areas are prone to mutual transformation from mild to moderate and then moderate to mild desertification. This indicates that when in areas with severe desertification, natural factors will have a strong influence on conditions while human activities can accelerate or slow down the development trends of desertification in areas with weak desertification. Mildly and moderately desertified land increased by one grade before 2000, and decreased after 2000, which is consistent with the change trend of desertified area.

In the long history of China, the country has always experienced periods with a dry climate system and wet interglacial stages with variations. The desertification of lands in arid and semi-arid areas has experienced volatility in response to climate change. In the study of nearly 5000 years, desertification and climate change have shown historical periods of a reversal of desertification, with a synchronization of changes in in climate and temperature. The desertification process in northern China in the last 5000 years has been influenced by the fluctuations in climate with the new ice age^51,52^. The Mu Us Sandy Land is located in the northwestern margin of the monsoon region of China, and its climate and environmental changes are closely related to the Southeast Asian monsoon which moves northward and retreats eastward and southward. When the East Asian summer monsoon intensifies, the climate is warm and humid, precipitation increases, chemical weathering intensifies, vegetation coverage increases, wind–sand activity weakens, and quicksand tends to be stabilized. When the East Asian winter wind intensifies, the climate becomes cold and dry, precipitation decreases, vegetation cover declines, wind–sand-related activities prevail, and wind–sand geomorphology develops^53,54^. This pattern of climate change and sand evolution is a response to changes in the winter and summer monsoon in East Asia^55^.

The pattern of change for desertified lands in the Mu Us Sandy Land is the result of a combination of natural and human factors. Vegetation coverage is an important factor affecting the development of desertified land. A large number of studies have shown that the factors affecting the growth of surface vegetation are mainly reflected in temperature and precipitation^33,56^. From Fig. 7, it can be seen that from 1990 to 2010, the temperature of the Mu Us Sandy Land showed a trend of gradual increase while precipitation showed a trend of slow decline, resulting in strong surface evaporation, water dissipation, and a reduction of available soil moisture. Drought affected the growth of vegetation and promoted the development of desertification. After 2010, the climate became warm-wet, which played a positive role in the reversal of desertification. Under the general background of climatic warming, the overall increase in temperature and the uncertainty of precipitation will strongly influence the way in which desertification develops. In addition, as wind speed is the main driving force for the development of desertification, it plays an important role in the process of surface erosion, transportation, and accumulation of sand materials^57,58^. Li concluded that from 1975 to 2017, wind speed, sand-driving wind speed, sand-driving wind frequency, and sand-transporting potential all showed a downward trend in the Mu Us Sandy Land, and the overall sand-transporting capacity had declined significantly^35^. Zhu believed that when the wind speed in April to July was lower than 2.46 m s^−1^, the FVC of the Mu Us Sandy Land would increase by 5%; Zhu also believed that wind speed was the climatic factor that had the greatest impact on vegetation growth and FVC^59^. The natural factors change cycle is longer and the range of change is relatively small, so, human interference can affect the dynamic trend of desertification in a short period of time. The uneven population distributions in the Mu Us Sandy Land, with more people in the east and fewer in the west, and the increasing population have caused human activities to aggravate the change of land structure. These include the unreasonable and unsustainable use of land resources, intensive land use, excessive woodcutting, and a failure to carry out vegetation restoration after land was abandoned. In recent years, the open-pit coal mining in the Mu Us Sandy Land has created very serious problems. If open-pit coal mines are not restored promptly, this will threaten to increase the intensity of land desertification. The impact of changes in land use structure on the desertification process is also very important. The impact of other human factors such as land use structural adjustment and policy measures related to the process of desertification is also very important. The environment deteriorated from 1990 to 2000 (Fig. 9). Since 2000, the policies of returning farmland to forest (grass), afforestation by aerial seeding, closure of sandy areas to grazing, and the banning of rotational grazing have been implemented in the Mu Us Sandy Land. In addition, the law of the People’s Republic of China related to the prevention and control of desertification was implemented in 2001, providing strong support for the control of desertification. From 2000 to 2013, the cumulative afforestation area of Mu Us Sandy Land reached 13,800 km^2^^60^, and the program had a significant effect in controlling erosion. Among the afforestation areas, the woodland area of Uxin Qi, located in the hinterland of the Mu Us Sandy Land, increased from 71 km^2^ to 203 km^2^ from 2000 to 2015, with an average annual growth rate of 7%^60^. At the same time, affected by the local “prohibition of grazing, rest grazing, rotational grazing”, the structure of livestock in pastoral areas has also changed, which mainly shows that the number of goats declined relatively while the number of sheep and pigs increased relatively, which further reduced the pressure on grasslands in the desert area^61^. Therefore, managers of the Mu Us Sandy Land should adjust the relationship between population and land based on the nature of the sandy land, so as to achieve the coordinated development of the economy and the environment.

## Conclusions

The dynamic process of change in land desertification over time in the Mu Us Sandy Land included the year 2000 as a turning point in three stages. A developmental stage from 1990 to 2000 when moderate and severe desertification primarily occurred, mainly in the western region of the Mu Us Sandy Land. The rapid reversal stage from 2000 to 2010 included a reversal of severe desertification at a rate of 9.7% × a^−1^, while mild desertification increased at a rate of more than 12.8% × a^−1^; meanwhile, desertification reversed in the northwestern part of the Mu Us Sandy Land. A steady reversal stage (2010–2017) occurred with primarily mild and moderate desertification while different levels of desertification reversed, although the speed of reversal was slower than the previous stage; the central and southern Mu Us Sandy Land area mainly remained state of stability and reversal. However, desertification has worsened in Shenmu County, north of Yuyang, and the eastern region with an overall level of desertification tending to decline.

The evolution rules of the spatial distribution of desertification showed that the gravity center of desertified land from east to west was mild, moderate, severe, extreme desertification, which is consistent with the transformation trend of the spatial distribution of desertified land in the Mu Us Sandy Land. By 2017, Uxin Qi, which is located in the hinterland of the Mu Us desert, experienced the most serious desertification. The areas of Otog Qian Qi, Shenmu County, Yuyang district, Yanchi County, and Ejin Horo Qi mainly experienced mild and moderate desertification. Moderate and severe desertification were the main types in Otog Qi. Dingbian County, Jingbian County, and Hengshan County to which ranges from mild, moderate, and non-desertified land.

In the past 30 years, the wind speed has decreased year by year at the rate of 0.1 m s^−1^, which directly reduced wind erosion and the soil transportation capacity of winds in the Mu Us Sandy Land and promoted the reversal of desertification. Between 1990 and 2010, the climate became warm-dry, and environmental protection policies and human efforts designed to prevent and control desertification played an important role in reversing desertification. Therefore, the control and improvement of conditions designed to address desertification in the Mu Us Sandy Land are the result of the joint action of local climate change and human activities. From 2010 to 2020, under the background of a warming-wetting climate, the rational use of resources in sandy areas and the strengthening of the scientifically-sound control of desertification-inducing factors are the keys to the currently observed reversal of desertification.

## Data Availability

All the data will be available upon motivated request to the corresponding author of the present paper.
